# Miscellaneous Cases

**Published:** 1852-03

**Authors:** L. P. Yandell

**Affiliations:** Louisville, Ky.


### WESTERN JOURNAL

OF

### MEDICINE AND SURGERY.

MARCH, 1852.

ART. I.—*Miscellaneous Cases.* By L. P. YANDELL, M. D., of Louisville, Ky.

*A Case of Anasarca.*—Miss E. V——, aged 22 years, of phlegmatic temperament, a native of Virginia, who had not enjoyed good health from her childhood, became dropsical about the age of puberty. Her father, who was about 50 years of age, was of the same temperament, indolent in his habits, and addicted to drink. Her mother was unhealthy, and suffered with ascites when pregnant with her first child, and with general dropsy during a subsequent pregnancy; of which she was relieved by a gentle mercurial course, followed by bitter tonics. One of the brothers of our patient died of dropsy, in Virginia, in the sixteenth year of his age, and all her brothers and sisters presented an anemic, relaxed, unhealthy appearance. She menstruated at the usual period, and with the irruption of the menses the hydropic symptoms became more prominent. Her health seemed still further to suffer from her change of residence, and the use of limestone water, which brought on obstinate diarrhea.

When the patient applied for advice, Oct. 1st, the dropsical effusion was universal; every part of the surface—the scalp, as well as the body and extremities, pitted on pressure. Her catamenia had not appeared for several months; she had suffered repeated attacks of hematemesis, and once had a slight hemorrhage from the lungs. These hemorrhages continued to recur at intervals of about a month during her illness. Her appearance was so indicative of ill health that no suspicion of pregnancy was excited by the suppression of her menses, and the repeated attacks of hematemesis were attributed to amenorrhea.

Calomel was prescribed, at night, in doses of 6 grains, to be followed by jalap next morning. At the same time she was to take carbonate of iron, 5 grains, three times daily, in combination with carbonate of potash. The calomel and jalap produced drastic purging but no reduction of the swelling followed, and after a few days this course was abandoned. The patient was now put upon a mercurial course. She took calomel, 1 grain, and opium ¼ grain, every night; and the muriate of mercury, 1-12 grain, three times daily. Under the use of these remedies the effusion began to subside, and she appeared to be convalescent. She continued to improve for several weeks upon a tonic course, which was combined with the mercurials; but from exposure to cold her symptoms became aggravated, and, conjoined with the others, she now presented those of hydrothorax. She was unable to assume a recumbent posture; she had a troublesome cough, accompanied by palpitation, and a distressing sense of suffocation. Her skin was dry; her urine scanty and high colored, without any appearance of coagulation by heat or nitric acid.

Dec. 1st. Patient's condition unimproved. Prescribed the deuto-chloride of mercury as before, until her gums should be slightly affected. Tinct. digitalis, 6 drops, three times daily, was also prescribed; and with these she took vegetable tonics, and under this treatment in a short time began to void large quantities of urine, by which the anasarca was sensibly diminished.

Jan. 1st. But little improvement in the case. The mammary glands are enormously distended, and the skin over them is affected with erysipelatous inflammation. According to the statement of the patient, her menses have now been absent thirteen months; but certain appearances about her begin to excite a suspicion that she may be pregnant. Emmenagogues, which had been directed, are to be discontinued. She is to persist in the use of the mercurial and the bitters. The effusion subsided for a time, but again returned.

April 1st. The condition of our patient is less favorable. The effusion is very great, rendering the recumbent posture nearly impossible; her breasts are excessively tense, and discharge a viscid, yellowish fluid; she is troubled with cough, and has occasional slight discharges of blood from the lungs, and a frequent recurrence of hematemesis. Her stomach is so irritable that she retains with difficulty the smallest quantity of food; she is pale and her countenance is anxious; her urine copious, limpid, and colorless; the alvine dejections scanty and clay-colored. Ordered bi-tart. potas., ʒi, rhubarb ℈j, to be repeated once every six hours, until copious purging is induced.

April 3d. The purgatives have acted freely, producing dark-colored, watery discharges, under which the patient appears to be recovering strength. Repeat them to-day.

April 5th. Patient much improved. Directed squills, ʒss, opium, 8 grains; to be made into thirty pills, of which give four daily, at intervals of four hours.

April 19th. The dropsical symptoms have disappeared rapidly. Prescribe a course of iron, bitters, and generous diet.

April 25th. She was seized with labor-pains, this morning, and gave birth to a child six weeks before its time. Found her agitated and excited in mind, with a flushed face; she has had several convulsions of a hysterical character, and is occasionally delirious. When roused, she complains of pain in the head and chest. Castor oil was given and its action promoted by enemas. In her exhausted situation bleeding was not resorted to. A blister was applied to the nape of the neck, and afforded relief to the cerebral symptoms.

April 30th. The hysterical convulsions continue to recur but with less violence; scarcely a trace of the hydropic affection remains, and her general health is improving. By the end of the month it was so far restored that she ceased to take medicine.

In several respects this was a remarkable case. Its duration was remarkable; she was the subject of the disease from about her fifteenth to her twenty-third year. It is an interesting feature in the case, that her mother was twice the subject of dropsy under similar circumstances, and that one of her brothers died of the same complaint. Its complication with pregnancy, which occurred after she had been for several years dropsical, and, according to her account, some months after the suppression of her catamenia; the hemoptysis, and the recurrence, almost monthly, of hematemesis; and, finally, the recovery of the patient under all these circumstances, render the case a very interesting one. In reviewing its history after the lapse of many years, I do not think I could say much in approbation of the plan of treatment pursued, which seems to have been directed without much system, and with no fixed views of the pathology of the disorder. What its pathology was, is indeed still a matter of conjecture. The patient had been cachectic from early girlhood; she was pale, anemic, of a flaccid fibre, and bloated all her life, and the hydropic effusion may have resulted from this relaxed condition of the system. Her liver at one time performed its function imperfectly, as was evidenced by the color of her stools; but it may be doubted whether hepatic derangement was at the bottom of the disorder. Remedies which afforded no relief at one stage of the disease, appeared to act favorably at a later period. Thus the purgation which failed to evacuate the fluid at first, was followed subsequently by free diuresis and reduction of the dropsical swelling. The cerebral symptoms after her confinement probably grew out of her anemic condition, as similar symptoms are known to follow a severe loss of blood, and would have been relieved or mitigated by the use of opiates.

*A Case of Intussusception.*—J. G——, aged twenty-one years, a farmer by occupation, thin, sallow, recently married, was attacked on the night of the 9th of February, with symptoms of colic, with which he had repeatedly suffered. The pain was incessant and very severe; his pulse was small but tense; his countenance expressive of great anguish. When I saw him next morning, the aperients which he had taken in the night had produced no effect. He was bled, and castor oil with laudanum was administered, at the same time that enemas were used freely. The bowels remaining unmoved, more drastic purgatives were resorted to and the clysters continued. He complained of acute local pain in the right iliac region, where a tumor could be felt, which was evidently the seat of the obstruction. He referred all his sufferings to this spot and urged me to remove the tumor by an operation. A large dose of calomel and opium was given, under the influence of which he slept, and in that situation I left him in the hope that a passage would be effected in due time.

Feb. 10th. My patient has passed a sleepless night, the pain returning in all its violence so soon as the effects of the opium passed off. His bowels are still unmoved, and the tumor is more palpable than it was yesterday. Croton oil was administered and recourse was again had to the enemata; but it was now remarked that only a very limited quantity of water could be forced into the bowels. His stomach became irritable, and he began to reject everything given. About 3 o'clock, P. M., the pain subsided, his pulse began to sink, his countenance became hippocratic, and in a few hours he died without further suffering.

*Autopsy.*—An examination of the body was made next day at the request of the friends of the deceased. On laying open the abdominal cavity the tumor which had been perceived in the right iliac region during the illness of the patient was at once discovered; and on examination proved to be an invaginated segment of the colon. The intussusception was *retrograde*, as it is technically expressed; that is, the lower part of the intestine was received into the upper. The invaginated portion, seven inches in length, was much swollen, thickened, dark, and sphacceleted. Tubercles, in the caseous stage, were found in the mesentery. No other cavity was examined, the cause of the malady and death having been made sufficiently manifest.

After the melancholy termination of this case, the question naturally arose in the mind whether an operation, so much insisted upon by the patient, would have been justifiable under the circumstances. All the authorities within my reach at the time were consulted with reference to the point; but I found nowhere any recorded instance of such an operation, nor did any writer recommend it in cases of the sort. The difficulty, in the first place, of recognising the existence of intussusception while the patient lives, and, in the next, the chance there is of removing the obstruction by other means, seemed to forbid a resort to so hazardous an expedient. Now and then, when all the measures tried have failed to effect an evacuation, the invaginated bowel has separated and been voided per anum, the patient recovering. The resources of nature in such circumstances are so great, that writers have recommended reliance upon them rather than recourse to a procedure never allowable in the earlier stages of the disorder, and of no avail when it has advanced to the stage of mortification.

Nevertheless, since the foregoing case transpired, this operation has been successfully performed, and, as the coincidence is somewhat singular, I may remark, in the neighborhood in which my patient resided. The surgeon who performed it was John R. Wilson, M. D., then of Rutherford, now of Davidson county, Ten. It is reported in the Transylvania Journal of Medicine, vol. 8. p. 486. The subject was a negro man, who was owned by Charles Dement. He had been for seventeen days affected with colic, in which time no alvine dejections had taken place; stercoraceous vomiting had come on, and there seemed to be no hope except in an operation. The evening before this was decided upon, after exhausting the list of cathartics, as a dernier resort some ounces of crude mercury were administered. The operation was performed towards the latter end of December, 1831, in the following manner: An incision was made along the linea alba, commencing above the umbilicus and extending two or three inches below it, being in all about five inches in length. The bowels being protruded through the wound, the portion involved in the stricture came at once into view. The invagination was found to be in the ileum. Adhesion had taken place between the surfaces in contact in consequence of the effusion of coagulable lymph, and considerable force was required to break up the attachments, so much, in fact, that there appeared to be danger of rupturing the coats of the intestines; but no such accident occurred. The portion of the bowel strangulated, though very much thickened, swollen, deeply congested, and of a dark, livid color, had not become gangrenous, and was consequently suffered to remain. The vessels of the omentum were engorged with black blood, apparently stagnant, and, like the intestine, this body seemed also on the verge of mortification. A cloth moistened in warm water was spread over the orifice during the operation, so as to exclude as much as possible the air from the cavity. The wound was closed by several stitches and adhesive strips, and the patient, soon after being put to bed, voided the mercury which he had taken the evening before. His recovery was rapid and complete.

The reporter of this case, Dr. W. W. Thompson, thinks that the result, under circumstances so unfavorable, warrants the propriety of the operation in similar cases. "In similar cases," certainly, the operation would be proper; but the difficulty is to determine at what period it has become necessary and yet not ceased to be availing. I should say that, in the case of my patient, there was a time when the operation might have been performed with fair prospects of success; and that the painful tumor felt in his side, and the impossibility of forcing more than a small quantity of fluid into his bowels, pointed to the existence of an impediment not likely to be overcome by medicine. In such a case, it strikes my mind that an operation would be justifiable.

*A Case of Epilepsy resulting from Fracture of the Cranium.*—J. A——, when a lad eighteen years of age, received a blow from a piece of falling timber, causing a fracture of the right parietal bone near the sagittal suture at its junction with the coronal. The fracture was not attended by depression of the bone or any alarming symptoms, and he recovered in a short time from the immediate effects of the injury; but after a few months he became epileptic. At first the convulsions recurred at long intervals, and his general health was so little impaired that he married in a year or eighteen months after receiving the injury. When I first saw him his general health was still good, and his intellect but little impaired, though the convulsions recurred with great regularity once in four weeks. Not many months after, in a violent fit, in which blood issued freely from his nose, he suddenly died. No post-mortem inspection was made at the time of his death, but four years subsequently his cranium was disinterred, and a very interesting pathological fact brought to light. Immediately under the fracture, the traces of which were distinctly visible, there was found growing from the inner table of the skull a spiculum of bone, an inch and a quarter in length, which must have pierced the brain, and, by the continual irritation produced by it, undoubtedly excited and kept up the convulsions. From the position of the abnormal growth, there is reason to believe that it caused ulceration and rupture of the walls of the superior longitudinal sinus, and thus gave rise to hemorrhage upon the brain and the sudden death.

The examination of the head of this individual was suggested by the operations performed by Prof. Dudley, many years since, in cases of epilepsy resulting from injuries of the brain, and illustrates very forcibly the truth of his theory and the propriety of his practice in such cases. There is good reason to believe, that trephining in this case would have been attended with success; and it is certain that it would have given the patient the only chance for health and prolonged life. Great as are the hazards of the operation, especially when it involves injury of the dura mater, no doubt can be entertained that it would be proper in such a state of things as existed here; and where epilepsy succeeds to fracture of the cranium, such or a similar condition of things may always be suspected.

*A Case of Inguinal Hernia Spontaneously relieved.*—Mrs. O——, aged thirty-six years, the mother of ten children, when in labor with her eleventh child, became the subject of inguinal hernia. Her children had been born in quick succession; she was the mother of six in five years from the time of her marriage, and with every one she had "a bad time." Her menses usually appeared in a month after her confinement, and, as a general rule, she had but one period. For several years each pregnancy, from the second month to the conclusion of it, was attended with distressing neuralgia. As a result of such frequent childbearing and so much suffering, her health had become much impaired.

It was supposed that an operation was necessary at the time I saw her, a week after the descent of the bowel; but on examination it was evident that, though there was much obstruction, complete strangulation had not taken place, and it was decided to trust to further efforts at reduction. The tumor was filled with fecal matter, and was tender to the touch. Castor oil was prescribed; enemas were freely used, and gradually the size of the tumor began to diminish, and its sensitiveness to disappear. In a few days nothing of it remained.

The taxis would in all probability have been followed by immediate relief, if it had been resorted to before the case had progressed so far; but at the time she was seen the distension and tenderness of the intestine were such, that it could not be practiced with efficiency. It may be a question whether it was proper, under such circumstances, to wait upon the action of such remedies as were employed—whether it was not our duty to have relieved the confined bowel by an operation? The issue here commended the course adopted; but it is doubtful whether most cases presenting a similar array of symptoms would have a similar termination.

*A Case of Poisoning by Rhus radicans.*—George, a healthy boy four years of age, was poisoned about the 20th of November with the *Rhus radicans.* The eruption presented itself chiefly on the face, which was covered with vesicles, and, very much swollen. In the end, superficial abscesses formed on his face, and about his ears. The pain occasioned by the eruption was much aggravated by the heat of the fire.

The *red wash* was prescribed over all the surface affected, and afforded prompt relief from the burning sensation in the skin. In a few days, under the use of this wash, with mild saline cathartics, the affection disappeared; but it is a curious and interesting feature in the case, that the eruption returned the ensuing autumn about the same time in the month. Whether it did so more than once I had not the opportunity to remark, as the father of the boy removed shortly after his second attack to a distant part of the State.

*A Case of Purpura Hemorrhagica.*—Miss O——, an unmarried lady, twenty years of age, whose health had previously been good, about the time of her monthly period became indisposed. I saw her on the 15th of March, at which time she had been suffering for six days with what she regarded as a profuse flow of her menses. Three days after the uterine hemorrhage came on, her gums began to bleed, the blood issuing from those of the right side. Her breath was fetid, bowels costive, pulse 120 and quick, countenance anxious. Prescribed calomel, ipecacuan as a nauseant, and spirits turpentine.

16th. No improvement in the case. Uterine hemorrhage continues, as also the bleeding of the gums, which are swollen and puffy; pulse small and frequent. Bowels moved twice; passages green. Ipecacuan operated once as an emetic. Directed a blister to be applied to the lower jaw; pills of cal. aloes and rhubarb; spirits of turpentine; sulph. quinine, 1 gr., every two hours.

17th. Patient worse. Face livid; cramp of stomach; uterine hemorrhage not arrested; bleeding from the gums less; pupil of one eye dilated; pulse failing. In the evening, had a recurrence of distressing pain in the stomach.

18th. Patient moribund. Face livid; tongue black. She died that evening.

This seems to have been a case of idiopathic purpura. No post-mortem examination was made.

Louisville, Feb. 16, 1852.

